# Greenhouse Gas Emissions from Beef Cattle Breeding Based on the Ecological Cycle Model

**DOI:** 10.3390/ijerph19159481

**Published:** 2022-08-02

**Authors:** Hongpeng Guo, Zixu Su, Xiao Yang, Shuang Xu, Hong Pan

**Affiliations:** College of Biological and Agricultural Engineering, Jilin University, 5988 Renmin Street, Changchun 130022, China; ghp@jlu.edu.cn (H.G.); suzx21@mails.jlu.edu.cn (Z.S.); yangxiao980618@163.com (X.Y.); xushuang_jlu@163.com (S.X.)

**Keywords:** life cycle assessment, ecological cycle, beef cattle breeding, greenhouse gas emissions

## Abstract

Over the past few decades, the supply of beef has increasingly become available with the great improvement of the quality of life, especially in developing countries. However, along with the demand for meat products of high quality and the transformation of dietary structure, the impact of massive agricultural greenhouse gas emissions on the environmental load cannot be ignored. Therefore, the objective of this study is to predict the annual greenhouse gas emissions of 10 million heads of beef cattle under both the ecological cycle model (EC model) and the non-ecological cycle model (non-EC model), respectively, in order to compare the differences between these two production models in each process, and thus explore which one is more sustainable and environmentally friendly. To this end, through the life cycle assessment (LCA), this paper performs relevant calculations according to the methodology of 2019 Refinement to the 2006 IPCC Guidelines for National Greenhouse Gas Inventories (2019 IPCC Inventories). The results have shown that the total GHG emissions of the non-EC model were almost 4 times higher than those of the EC model, and feed-grain cultivation and manure management were main emission sources in both models. The non-EC model produced significantly more emissions than the EC model in each kind of GHG, especially the largest gap between these two was in CO_2_ emissions that accounted for 68.01% and 56.17% of the respective planting and breeding systems. This study demonstrates that the transformation of a beef cattle breeding model has a significant direct impact on cutting agricultural GHG emissions, and persuades other countries in the similar situation to vigorously advocate ecological cycling breeding model instead of the traditional ones so that promotes coordinated development between planting industry and beef cattle breeding industry.

## 1. Introduction

At present, greenhouse gas (GHG) emissions have been already accepted as a global environmental issue and are widely considered a cause for concern by international society. According to the latest GHG inventory guidelines of Intergovernmental Panel on Climate Change (IPCC), the global net anthropogenic GHG emissions in 2019 were 59 ± 6.6 GtCO_2_-eq, about 12% (6.5 GtCO_2_-eq) higher than in 2010 and 54% (21 GtCO_2_-eq) higher than in 1990. In particular, approximately 22% (13 GtCO_2_-eq) of total net anthropogenic GHG emissions came from agriculture, which was the largest proportion after those of energy supply sector (34%) and industry (24%) [1,2,3], and this will lead to climate change that has consequences for oceans, weather, food sources and our physical health. However, although the gradual growth of the livestock industry has indeed met people’s urgent demand for high-quality meat to a large extent, it has also forced the environmental carrying capacity to be under unprecedented pressure.

Global GHG emissions from livestock increased by 51% from 1961 to 2010 because of increased demand for livestock products [4], and CH_4_ accounted for 50% of total emissions with 24% N_2_O and 26% CO_2_. In order to compare different animal products’ emission intensities, FAO (2021) calculated that meat from buffalo presents the highest emission intensity, with an average of 404 kg CO_2_-eq per kg of protein, followed by beef, with an average of 295 kg CO_2_-eq per kg of protein, whereas pork has much lower emission intensity, below 100 kg CO_2_-eq per kg of protein [5,6,7]. Moreover, beef and dairy cattle are the largest source of livestock emissions, i.e., 74% of global livestock emissions [4]. In 2019, CH_4_ emissions due to enteric fermentation of beef cattle represented by 14.9% of total GHG emissions from agriculture, whereas direct N_2_O emissions due to urine and gung represented by 2% in certain areas [8]. Therefore, it is necessary to calculate the GHG emissions in each process to explore a sustainable and environmentally friendly way to integrate crop and livestock.

LCA is a state-of-the-art methodology that estimates potential environmental impacts of products and totals, and evaluates the efficiency of resource use as well. It can be defined as the assembly and estimation of resource inputs and outputs ‘from cradle to grave’, including all up- and downstream activities over the entire life cycle [9,10,11]. According to previous studies, assessing environmental sustainability of production systems through life cycle assessment (LCA) to quantify GHG emissions has been a typical and extensively used method [12], especially in beef cattle production [13].

Fiore et al. (2018) [14] used LCA methodology to divide the GHG produced by Italy’s cattle farming into two major sources which were feed processing and production and enteric fermentation, contributing 45% and 39% of total emissions, respectively. Casey and Holden (2006) [15] focused on comparing typical Irish beef production system with five other production options and assumed the system boundary of Irish beef production includes four main processes, such as feed production, N fertilizer production, livestock manure management and electricity and diesel for agricultural operations, and thus obtained the range of GHG emissions from 7.17 to 11.26 kg CO_2_-eq per kg beef live weight per year so that estimated the potential effects of changing management to attain reduction in GHG emissions. However, estimation results are not exactly the same due to different regions, different system boundaries and different calculation parameters, etc. [16,17,18]. Nguyen et al. (2010) [19] concluded the global warming potential (GWP) per kg beef produced in the EU ranged from 16.0 to 27.3 kg CO_2_-eq, whereas the figure of 22.3 kg CO_2_-eq reported by Cederberg and Stading (2003) [20] and 20 kg CO_2_-eq by Adrian et al. (2006) [21]. Dramatically, the figure from Ogino et al. (2007) [22] seemed rather high compared to those mentioned studies (36.4 kg CO_2_-eq), and the reason was that it defined the retail beef yield percentage as 40% and included both the cow-calf and the fattening system.

Concerning reducing GHG emissions from livestock, especially from beef cattle, different works provide targeted corresponding measures from various angles via the local condition and general emission situation of each process, which can serve as references for other regions with similar characteristics of beef cattle breeding. Johnson et al. (2003) [23] confirmed that intensive grazing not only reduces GHG emissions by 16%, but also improves profitability by 13%. A study by Mogensen et al. (2012) [24] considered that if the individual reduced their consumption of meat to approximately 100 g, this would reduce both the emission of greenhouse gases and the nutrient losses considerably (by, respectively, 25 and 31%), so changing diets is potentially one of the most powerful ways of mitigating GHG emissions. There are also scholars who concentrate on adjusting feed ingredients to improve ruminant livestock weight gain with GHG emissions reduction. For example, Ridoutt et al. (2022) [25] suggest that feed supplementation with Asparagopsis taxiformis has the potential to reduce the sector’s carbon footprint by 1~4% in 2030. This finding is precisely based on the comparison of GHG emissions of those emission reductions that make it possible to better identify which measures are more suitable for specific regions, or even for achieving national goals of agricultural carbon-mitigation. Ruviaro et al. (2015) [26] analyzed a case study of a farm in southern Brazil and found production systems achieved the lowest CO_2_ emissions and the highest feed conversion rate with dry matter intake digestibility (DMID) from 52 to 59%, generating lower CH_4_ and N_2_O emissions per production system. Southern Brazil has committed to developing beef cattle production with efficiency and sustainability. There are also the Low Carbon Agriculture program and the cattle beef development program of Santa Catarina, which encourage investments in sustainable technologies for GHG mitigation, such as biological nitrogen fixation, integrated crop-livestock systems, and so on [27]. Furthermore, integrating dairy and beef production would enable the New Zealand beef sector to reduce annual GHG emissions from beef production by almost 22% of the total sector’s emissions [28,29]. Indeed, virtuous cattle farms present a planned resource recycling system allowing for low GHG emissions [14].

On the other hand, the idea of integrating planting and breeding systems has gradually developed. Recycling crop residues and organic manure as feeds for livestock and soil amendments, is a kind of resource-efficient benefit of crop-animal integration that might be worth using for other countries [30]. Cerutti et al. [31] compared the differences between three production practices (conventional, organic and integrated) in order to quantify the climate change reduction potential of different production methods. Through LCA analysis, they found that integrated production showed the best performance in reducing GHG emissions per unit of product, mainly due to the increased productivity of the integrated system, while conventional production practices had higher GHG emissions than both organic and integrated production practices. Takacs et al. [12] conducted a systematic review to examine the effectiveness of life cycle-based interventions in improving the sustainability of production systems, concluding that integrated production systems should be preferred instead of conventional high inputs and outputs systems. The ecological cycle is oriented to the combination of planting and breeding, the cycle of agriculture and animal husbandry and green development, while simultaneously realizing comprehensive utilization of straws and transformation from manure to fertilizer [32]. Most importantly, the integrated production is the core of this pattern. This kind of model is one of China’s major measures to tackle carbon emissions through ensuring resources recycling between planting industry and beef cattle breeding industry. Although the share of China’s livestock sector in total agricultural GHG emissions significantly declined from 47.13% in 2009 to 43.61% in 2019, there has been a lot of room to improve [33,34]. In addition, as a potent tool whose methodology will have a distinct effect on the selection of mitigation measures and show the effect of these measures, the latest emission inventory reports with up-to-date parameters and data should be selected and used [35].

This study takes Jilin Province in China as an example, thereby aiming to quantify the GHG emissions of 10 million heads of beef cattle under the ecological cycle model (EC model) and non-ecological cycle model (non-EC model) to show the effects of different mitigation measures, according to the life cycle assessment (LCA). The second is to compare the differences in each process of GHG emissions between both breeding models and explore which one is more sustainable and environmentally friendly. Moreover, the results can also provide theoretical reference for the ecological cycle of agriculture in other regions with similar breeding characteristics worldwide.

## 2. Materials and Methods

### 2.1. Study Subject Profile and Data Source

Jilin Province, located in the central part of northeast China, is a main corn-producing area in China, spanning 40 52’~46 18’ north latitude, which is in the same one as both the U.S. Corn Belt and the Ukrainian Corn Belt, and together they are known as “Three Golden Corn Belts” [36,37]. The characteristics of the popularity of the corn cultivation in these regions are attributed to favorable climatic conditions [38]. However, since the beginning of ancient agricultural civilization in Jilin Province, the long-term use of traditional agricultural technology and processing techniques has been unable to include reuse and recycling of resources between planting and breeding systems, which has resulted in problems such as the disconnect between agriculture and livestock, the surplus of straw, agricultural pollution from non-point, and so on.

This paper assumes that the breeding stock of beef cattle in the province includes 10 million heads and the average weight of beef cattle is 319 kg (IPCC default weight) with 365 days of fattening period, via an intensive fattening method. According to the field investigation and related information, the amount of mixed concentrate feed required for beef cattle fattening has been determined by the fattening degree and farmer’s seasonal harvest and income level, etc., while it is approximately 2.5~6.5 kg·head^−1^·d^−1^ in general. There is no significant difference in the basic ingredients of the concentrate feed and the proportion of each ingredient over time, so this article did not compare differences of concentrate feed under these two models. Based on the survey of Jilin Province, the vast majority of beef cattle farmers have still continued to separate planting and breeding systems and regard the straw of common corn as coarse fodder after crushing by machines, even feeding directly without any processing.

Additionally, the input amount of coarse fodder is usually about 8~10 kg·head^−1^·d^−1^, while alfalfa, dry rice straw, wine lees, wheat straw, peanut stalks, etc., can be mixed in the feed at the same time, although there are very few large-scale farms which will add a certain proportion of ensile corn. For the sake of facilitating the calculation, we only consider the consumption of different corn as the difference between EC model and non-EC model in terms of roughage, namely, beef cattle need 20 kg·head^−1^·d^−1^ of silage corn under EC model and 9 kg·head^−1^·d^−1^ of common corn under another model. Therefore, it can be calculated that annual feed consumptions of 10 million heads of beef cattle in two models are 7.30 × 10^7^ t and 3.29 × 10^7^ t for silage corn and common corn, respectively.

The structure of corn straw utilization in Jilin Province consists of 9.88% for feed production, 61.60% for household burning and 24.15% for waste and incineration [39,40]. We combine the latter two into a single because both of them are essentially direct combustion, i.e., straw burning accounts for 85.75%, and other proportions of straw utilization are neglected because of their small percentage. In this case, this paper supposes feed production of beef cattle is equal to food consumption, and the amount of feed production and straw burning include total corn straw consumption required for non-EC model (31.80 × 10^10^ kg). Depending on interviews with locals, we found that fresh whole silage corn per acre could produce 0.9 t straw, in comparison with common corn (0.5 t), thus obtaining planting areas of both models: 9.01 × 10^5^ hm^2^ for silage corn and 70.66 × 10^5^ hm^2^ for common corn. Manure per beef cattle, including dung and urine, was set to 22.67 kg·d^−1^, consequently obtaining 8030.00 kg of manure excreted by each beef cattle annually [4,41,42].

Furthermore, this study assumes that the rate of manure returned to the field is 100% in the EC model, which indicates manure completely consumed and locally utilized. In contrast, in the non-EC model, composting is the usual way of manure management for most farmers in Jilin Province, and some of it even discharged directly without any processing, despite just 45% entering the planting system as fertilizer [43]. The methodology of emission evaluation and various emission factors in this paper referred to 2019 IPCC Inventories, and other data are derived from two different yearbooks except other scholars’ scientific works: the China Statistical Yearbook and the Jilin Province Statistical Yearbook [44,45].

### 2.2. System Boundary

The system boundary of this study included entire processes of the two models from the planting system to breeding system, involving emission activities associated with the production processes of the complicated system. Additionally, because of differences among different regions, in order to be convenient for computation and comparison, the system boundary was defined by several key processes which could be properly representative of local characteristics as much as possible, since site-specific data are necessary to precisely describe each process in specific region [46]. Thus, there are two system boundaries, one is for the EC model and another is for the non-EC model.

For the purpose of simplifying operation, irrigation, transportation, and energy consumption, etc., are not appropriate indicators of environmental impacts in several instances [47,48,49], they have to be considered outside the system boundary of this study.

**Figure 1:**
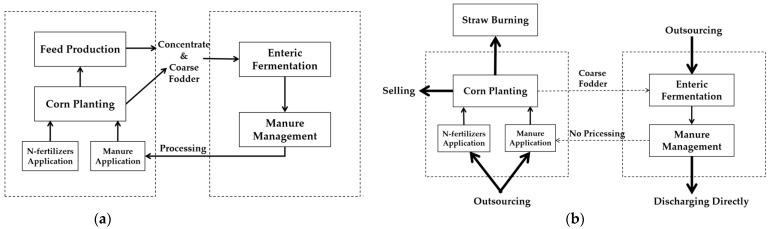
(**a**) The EC model; (**b**) The non-EC model. Note: (1) Each solid line box indicates an independent process, and each dotted line box just indicates that the processes in the box occur in the same place; (2) In (**b**), each thick solid line arrow indicates the largest percentage of resources circulate in that channel, the thin one comes second, and the dotted one is the lowest.

Hence, in terms of the system boundary of the EC model, it includes corn planting, N-fertilizers application, manure application, feed production, enteric fermentation and manure management. The counterpart (non-EC model) also has six processes which are corn planting, N-fertilizers application, manure application, straw burning, enteric fermentation and manure management. The system boundaries of the two models are as follows (Figure 1):

### 2.3. GHG Emission Calculation

CO_2_, CH_4_ and N_2_O are three primary GHG in agriculture [50], and they are converted to carbon dioxide equivalent (CO_2_-eq) using the global warming potential (GWP).

#### 2.3.1. CO_2_ Emissions from Corn Planting

In this subsection, we only calculate CO_2_ emissions generated during corn planting of silage corn by comparison with common corn required in roughage, as shown in Equation (1):

(1) $$E_{farm,i}=Q_{i}\times ef_{farm-corn}$$

$E_{farm,i}$ is the amount of CO_2_ emissions from planting corn in EC model or non-EC model, kg·a^−1^ (“a” is equal to “year”); i=1,2 indicates EC model and non-EC model respectively; $Q_{i}$ is the annual feed consumption of 10 million beef cattle in each model, kg·a^−1^; $ef_{farm-corn}$ is the CO_2_ emission factor that represents growing corn-based feed grain crops, t·t^−1^.

#### 2.3.2. N_2_O Emissions from N-Fertilizers Application

N_2_O emissions from N-fertilizers application consist of direct emissions and indirect emissions, and the indirect ones specifically from volatilization of NH_3_-N and NO_x_-N and leaching or run-off from N-fertilizers. The formula is as follows:

(2) $$E_{NF,i}=T_{NF,i}\times \left(ef_{ND}+ef_{NH}\times F_{GAS}+ef_{NL}\times F_{L}\right)\times \frac{44}{28}\times GWP_{N_{2}O}$$

$E_{NF,i}$ is the amount of N_2_O emissions (in CO_2_-eq) from N-fertilizers application in the *i* model, kg·a^−1^; $T_{NF,i}$ is the annual N-fertilizers application for corn planting in the *i* model, kg·a^−1^; $ef_{ND}$ is the direct N_2_O emission factor from N-fertilizers application, t·t^−1^; $ef_{NH}$ is the indirect N_2_O emission factor from atmospheric deposition of nitrogen on soils and water surfaces, t·t^−1^; $ef_{NL}$ is the indirect N_2_O emission factor from nitrogen leaching and runoff, t·t^−1^; $F_{GAS}$ is the fraction that is lost by volatilization in N-fertilizers application as NH_3_-N and NO_x_-N, t·t^−1^; $F_{L}$ is the fraction that is lost by leaching or run-off in N-fertilizers application, t·t^−1^; 44/28 is the conversion of N_2_O-N emissions to N_2_O emissions; $GWP_{N_{2}O}$ means 1 ton of N_2_O equals 265 tons of carbon dioxide (100a) [51].

#### 2.3.3. N_2_O Emissions from Manure Application

Detailed information can be seen in Equation (3).

(3) $$E_{NM,i}=T_{NM,i}\times \left(ef_{NDM}+ef_{NH}\times F_{GASM}+ef_{NL}\times F_{LM}\right)\times \frac{44}{28}\times GWP_{N_{2}O}$$

$E_{NM,i}$ is the amount of N_2_O emissions (in CO_2_-eq) from manure application in the *i* model, kg·a^−1^; $T_{NM,i}$ is the annual manure application for corn planting in the *i* model, kg·a^−1^; $ef_{NDM}$ is the direct N_2_O emission factor from manure application, t·t^−1^; $F_{GASM}$ is the fraction that is lost by volatilization in manure application as NH_3_-N and NO_x_-N, t·t^−1^; $F_{LM}$ is the fraction that is lost by leaching or run-off in manure application, t·t^−1^.

#### 2.3.4. CO_2_ Emissions from Feed Production

Generally, it is common to use conventional techniques and have nothing to process grain into feed in non-EC model, which leads to failure to become self-sufficient and mainly purchasing feeds from outside. Therefore, this process is only included in the EC model.

(4) $$E_{feed}=Q_{s}\times ef_{feed-corn}$$

$E_{feed}$ is the amount of CO_2_ emissions from silage feed production, kg·a^−1^; $Q_{s}$ is the annual feed consumption of silage corn to breed 10 million beef cattle, kg·a^−1^; $ef_{feed-corn}$ is the CO_2_ emission factor from silage feed production, t·t^−1^.

#### 2.3.5. GHG Emissions from Straw Burning

In the EC model, one of the objectives is to use the corn straw as resources and the utilization rate is up to 100%, which means that open burning of corn straw is thoroughly avoided. On the other hand, around 85.75% of corn straw is attributed to direct burning as household burning and waste incineration for lack of the reutilization techniques. As a result, this process only applies to the non-EC model.

(5) $$E_{burn}=E_{burn-CO_{2}}+E_{burn-CH_{4}}+E_{burn-N_{2}O}$$

(6) $$E_{burn-CO_{2}}=Q_{straw}\times R_{burn}\times G\times ef_{burn-CO_{2}}$$

(7) $$E_{burn-CH_{4}}=Q_{straw}\times R_{burn}\times G\times ef_{burn-CH_{4}}\times GWP_{CH_{4}}$$

(8) $$E_{burn-N_{2}O}=Q_{straw}\times R_{burn}\times G\times ef_{burn-N_{2}O}\times GWP_{N_{2}O}$$

$E_{burn}$ is the amount of GHG emissions (in CO_2_-eq) from straw burning, kg·a^−1^; $E_{burn-CO_{2}}$ is the amount of CO_2_ emissions from straw burning, kg·a^−1^; $E_{burn-CH_{4}}$ is the amount of CH_4_ emissions (in CO_2_-eq) from straw burning, kg·a^−1^; $E_{burn-N_{2}O}$ is the amount of N_2_O emissions (in CO_2_-eq) from straw burning, kg·a^−1^; $Q_{straw}$ is the annual amount of straw required in the non-EC model, kg·a^−1^; $R_{burn}$ is the straw burning rate, taken as 85.75% [39]; G is the straw burning emission factor, taken as 0.1 [5]; $ef_{burn-CO_{2}}$ is the CO_2_ emission factor of straw burning, kg·kg^−1^; $ef_{burn-CH_{4}}$ is the CH_4_ emission factor of straw burning, kg·kg^−1^; $ef_{burn-N_{2}O}$ is the N_2_O emission factor of straw burning, kg·kg^−1^; $GWP_{CH_{4}}$ means 1 ton of CH_4_ equals 28 tons of carbon dioxide (100a) [51].

#### 2.3.6. CH_4_ Emissions from Enteric Fermentation

Detailed information can be seen in Equation (9).

(9) $$E_{EF}=N_{a}\times ef_{EF}\times GWP_{CH_{4}}$$

$E_{EF}$ is the amount of CH_4_ emissions (in CO_2_-eq) from enteric fermentation of beef cattle, kg·a^−1^; $ef_{EF}$ is the CH_4_ emission factor from enteric fermentation of beef cattle, kg·head^−1^·a^−1^; $N_{a}$ is the annual number of head of beef cattle, head·a^−1^.

#### 2.3.7. CH_4_ Emissions from Manure Management

Detailed information can be seen in Equations (10) and (11).

(10) $$E_{MMC,i}=N_{a}\times VS_{i}\times MS_{i}\times ef_{MMC,i}\times GWP_{CH_{4}}$$

(11) $$VS_{i}=VS_{rate,i}\times \frac{W_{default}}{1000}\times 365$$

$E_{MMC,i}$ is the amount of CH_4_ emissions (in CO_2_-eq) from manure management of beef cattle in the *i* model, kg·a^−1^; $VS_{i}$ is the annual average volatile solids ($VS_{}$) excretion per head of beef cattle in the *i* model, kg·head^−1^·a^−1^; $VS_{rate,i}$ is the default $VS_{}$ excretion rate in the *i* model, kg·kg^−1^·d^−1^; $MS_{i}$ is the fraction of total annual $VS_{}$ for beef cattle that is managed in the *i* model, %; $ef_{MMC,i}$ is the CH_4_ emission factor from manure management in the *i* model, kg·kg^−1^; $W_{default}$ is the default average mass for beef cattle, kg.

#### 2.3.8. N_2_O Emissions from Manure Management

Detailed information can be seen in Equations (12)–(18).

(12) $$E_{MMN,i}=E_{MMDN,i}+E_{MMIDN-volatilize,i}+E_{MMIDN-leach,i}$$

(13) $$E_{MMDN,i}=N_{a}\times Nex\times MS_{i}\times ef_{MMDN,i}\times \frac{44}{28}\times GWP_{N_{2}O}$$

(14) $$Nex=N_{rate}\times \frac{W_{default}}{1000}\times 365$$

(15) $$N_{volatilize,i}=N_{a}\times Nex\times MS_{i}\times F_{GAS,i}$$

(16) $$E_{MMIDN-volatilize,i}=N_{volatilize,i}\times ef_{MMIDN-volatilize}\times \frac{44}{28}\times GWP_{N_{2}O}$$

(17) $$N_{leach,i}=N_{a}\times Nex\times MS_{i}\times F_{LEACH,i}$$

(18) $$E_{MMIDN-leach,i}=N_{leach,i}\times ef_{MMIDN-leach}\times \frac{44}{28}\times GWP_{N_{2}O}$$

$E_{MMN,i}$ is the amount of N_2_O emissions (in CO_2_-eq) from manure management in the *i* model, kg·a^−1^; $E_{MMDN,i}$ is the amount of direct N_2_O emissions from manure management in the *i* mode1, kg·a^−1^; $E_{MMIDN-volatilize,i}$ is the amount of indirect N_2_O emissions due to volatilization of *N* from manure management in the *i* mode1, kg·a^−1^; $E_{MMIDN-leach,i}$ is the amount of indirect N_2_O emissions due to leaching and runoff from manure management in the *i* mode1, kg·a^−1^; Nex is the annual average *N* excretion per head of beef cattle, kg·head^−1^·a^−1^; $ef_{MMDN,i}$ is the direct N_2_O emission factor from manure management in the *i* model, kg·kg^−1^; $N_{rate}$ is the default *N* excretion rate per 1000 kg of beef cattle mass,, kg·kg^−1^·d^−1^;$N_{volatilize,i}$ is the amount of manure nitrogen that is lost due to volatilization of NH_3_ and NO_X_ in the *i* model, kg·kg^−1^; $N_{leach,i}$ is the amount of manure nitrogen that is lost due to leaching in the *i* model, kg·a^−1^; $F_{GAS,i}$ is the fraction of managed manure nitrogen for beef cattle that volatilizes as NH_3_ and NO_X_ from the manure management in the *i* model, %; $F_{LEACH,i}$ is the fraction of managed manure nitrogen for beef cattle that is leached from the manure management in the *i* model, %; $ef_{MMIDN-volatilize}$ is the indirect N_2_O emission factor from atmospheric deposition of nitrogen on soils and water surfaces as NH_3_-N and NO_X_-N, kg·kg^−1^; $ef_{MMIDN-leach}$ is the indirect N_2_O emission factor from nitrogen leaching and runoff, kg·kg^−1^.

#### 2.3.9. GHG Emissions from the System Integrated Planting and Breeding

Detailed information can be seen in Equations (19) and (20).

(19) $$E_{T,i=1}=E_{farm,i=1}+E_{NF,i=1}+E_{NM,i=1}+E_{feed}+E_{EF}+E_{MMC,i=1}+E_{MMN,i=1}$$

(20) $$E_{T,i=2}=E_{farm,i=2}+E_{NF,i=2}+E_{NM,i=2}+E_{burn}+E_{EF}+E_{MMC,i=2}+E_{MMN,i=2}$$

$E_{T,i}$ is the total amount of GHG emissions (in CO_2_-eq) from the system integrated planting and breeding, kg·a^−1^.

### 2.4. Parameter Selection and Description

As different regions have different characteristics of the natural environment and breeding patterns, it is necessary to use parameter indicators with local characteristics in order to accurately predict local GHG emissions. In other words, we have to select some parameters from local data in this paper. In this circumstance, despite the difficulty of collecting data, we searched many studies and averaged a set of numbers if they represented the same parameters in other papers. Detailed information can be seen in Table 1.

## 3. Results

### 3.1. Estimation Results of Each Process in Different Models

Through calculation (Table 2), the total GHG emissions (in CO_2_-eq) of the EC model and the non-EC model were 19.62 × 10^7^ t·a^−1^ and 75.95 × 10^7^ t·a^−1^, respectively, whereas there was about 56.33 × 10^7^ t·a^−1^ difference between them and the latter’s emissions were nearly four times higher than those of the former.

Apart from this, the total GHG emissions from the planting industry were 11.23 × 10^7^ t·a^−1^ (57.24%) and 48.93 × 10^7^ t·a^−1^ (64.42%), with the total GHG emissions from the breeding industry of beef cattle being 8.39 t·a^−1^ (42.76%) and 22.83 t·a^−1^ (30.06%). 5.52% of GHG emissions came from straw burning in non-EC model, which indicates that the EC model can significantly limit GHG emissions compared with the non-EC model in both planting and breeding industries.

**Figure 2:**
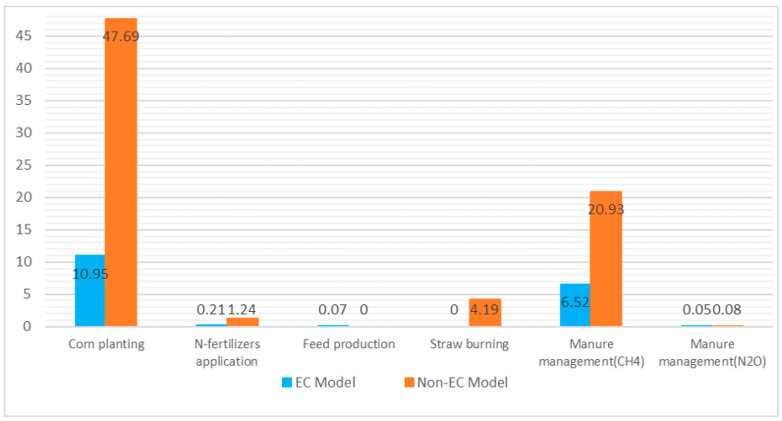
Bar chart of GHG emissions from the key processes in two models.

It was reckoned that the process of corn planting was the major source of GHG emissions in both models, 10.95 × 10^7^ t·a^−1^ and 47.69 × 10^7^ t·a^−1^, respectively, and they were responsible for 55.81% and 62.79%. Moreover, the gap between these two models remained significant in manure management that non-EC model created 14.44 × 10^7^ t·a^−1^ more GHG emissions than the EC model. The proportions of N-fertilizers application from both two models were extremely small and N_2_O emissions from non-EC model were 1.03 × 10^7^ t·a^−1^ higher than those of EC model. On the other hand, only in the processes of feed production and manure application, the GHG emissions produced by the EC model were higher than its counterparts. Owing to feed processing equipment, the EC model emitted 0.07 × 10^7^ t·a^−1^ of CO_2_, while the difference in manure application was quite small (27.24 t of N_2_O). The differences between these two models are shown in Figure 2.

### 3.2. Estimation Results of Different Types of GHG Emissions

Regarding CO_2_ emissions, as shown in Table 3, the single biggest difference between two models (40.63 × 10^7^ t·a^−1^), 11.02 × 10^7^ t·a^−1^ and 51.65 × 10^7^ t·a^−1^ produced by the EC model and the non-EC model, which accounted for 56.17% and 68.01%, respectively, of each planting and breeding system. CH_4_ emissions were the second biggest source of GHG emissions after CO_2_ emissions that non-EC model accumulated 14.59 × 10^7^ t·a^−1^ higher compared to EC model, with 42.51% and 30.19% of their respective total emissions. As for N_2_O emissions, whether in the difference between emissions of different models or in the proportion of corresponding systems, it seemed almost identical in these two models and both these values were small, for instance, the difference was only 1.11 × 10^7^ t·a^−1^ in terms of total emissions of N_2_O.

Furthermore, CO_2_ emissions were mainly created in the process of corn production in these two models, followed by straw burning, which was one of unique features of the non-EC model and accounted for 5.21% of the total emissions in this model. Therefore, it is crucial that adopting an appropriate irrigation system and using agricultural plastic films and fossil fuel resources, etc., in a reasonable way during production activities to reduce CO_2_ emissions instead of straw burning.

Most CH_4_ emissions generated from manure management, 33.23% of GHG emissions in EC model, compared with 27.56% in non-EC model. Although methane emissions from enteric fermentation of beef cattle in both models were 1.82 × 10^7^ t·a^−1^, EC model and non-EC model were responsible for 9.28% and 2.39%, respectively. From a different perspective, it is possible to reduce CH_4_ emissions by means of improving manure management.

The processes of manure application, manure management and straw burning affected the amount of GHG emissions, while nitrogen fertilizer application was the most important determinant of N_2_O emissions, and the emissions produced by this process accounted for 1.07% and 1.63% of total GHG emissions in each model. This implies that using fertilizers appropriately in corn planting is a suitable alternative to curb N_2_O emissions to some extent.

### 3.3. Uncertainty Analysis of Estimation Results

Although there are so many complicated reasons leading to estimation results’ uncertainties in various studies, the uncertainty is attributed to three major points in this study.

Firstly, the representativeness of the data involved in this paper was relatively limited because comprehensive data on the crop–livestock system in Jilin Province was so lacking and outdated that it required the expert consultation and field research to obtain. Moreover, we only chose those emission factors with high universality, which means those would be suitable for other regions and weaken local characteristics at the same time. In particular, the factors of transportation, processing, irrigation and energy consumption, etc., failed to be considered, which would inevitably have an influence on the results to some extent. On the other hand, the GWP values given in different years of IPCC reports were not exactly the same, consequently reducing the certainty of estimation results.

## 4. Discussion

### 4.1. Corn Planting Process

CO_2_ emissions from feed grain production are the most significant source of GHG emissions in the panting–breeding system.

In this process, the huge gap of GHG emissions between the two models (36.74 × 10^7^ t) is based on the fact that most farmers in Jilin Province have regarded traditional planting techniques as the main method throughout the cultivation of feed grains. Local farmers fail to have a full understanding of the advantages of whole plant corn silage, thus they are engaging in planting common corn rather than silage corn, which can reduce the richness and the diversity of the bacterial community and the protozoa number in the rumen of beef cattle, leading to a decrease in CH_4_ emissions during the enteric fermentation process [62]. Additionally, by the above calculation, the planting area of common corn tends to be eight times bigger than that of silage corn. It means it is enough for planting silage corn to use much smaller planting areas and offer much higher yields to satisfy the same number and heavyweight of beef cattle as common corn demanded, meanwhile generating lower GHG emissions.

The conventional production uses a lot of energy, fertilizers and pesticides, etc., during corn planting process, generating higher GHG emissions than the integrated system [31]. Moreover, Jilin Province has been depending on manpower and animal power, at the same time, generally relying on farmers’ personal experience to determine the use and times of plowing, fertilizers, pesticides, agricultural plastic films and agricultural machinery to a certain extent. Therefore, the planting system in Jilin Province is a typical non-EC model. Under this circumstance, it has to require a higher farmer quality. By contrast, in EC model, large-scale farms generally maintain long-term communication and close cooperation with agricultural universities and research institutions in order to keep pace with advanced planting-breeding techniques and modern facilities. It is also a low input/output system that use fewer resources and therefore generates lower GHG emissions [46,63]. In addition, it tends to rotate a variety of crops, stagger the sowing and harvesting times of different crops, greatly improve the efficiency of the use of agricultural machinery and increase production, in particular, achieve energy saving and emission reduction [64].

### 4.2. Manure Management Process

In the EC model, organic fertilizers and residues are produced through aerobic composting after multiple processes that effectively reduce GHG emissions, and then these substances can be locally absorbed into soil. However, in the non-EC model, as the opposite, the technical means of manure management are more traditional and outdated. This means that manure waste is often discharged directly to the farmland without processing and the comprehensive utilization rate is less than 60%. This treatment method would be a barrier to resource recycling in the planting–breeding system, meanwhile, it generates more than three times GHG emissions compared to that of EC model. Inevitably, both conventional and integrated systems produce a large proportion of GHG emissions in manure management process. In this case, some specific approaches to reduce both CH_4_ and N_2_O emissions during this process, including anaerobic digestion of livestock manure and optimal timing of manure application within the growing season [65].

Therefore, it proves that the way of manure management has a deep influence on GHG emissions and farmland comprehensive productive capacity, whereas the EC model is a more effective method to promote planting–breeding combination and develop circular agriculture.

### 4.3. Straw Burning Process

Straw burning is a relatively inefficient treatment method of corn stalk residues.

The reason that corn straws are burnt in fields after harvest is that farmers tend to remove the straws from fields quickly and save the cost of transportation and the storage of the straws [66]. As the main method of the non-EC model, open burning of corn straw has caused serious environmental pollution, namely, more than 4.19 × 10^7^ t GHG emissions per year produced by the non-EC model compared to the EC model.

Jilin Province is the major agricultural province in China, especially corn straw resources are extremely rich, such as straws of rice, wheat, corn, barley, millet, sorghum, legume, cotton, sugarcane, and so on. Nevertheless, the contribution of emissions from corn straw is almost the greatest (37–49%) [67]. Even though burning straw appropriately can facilitate sowing seeds, leaching of abundant biotic potassium in the plant ash and killing pests with its eggs and so on. It is not unrealistic that the large-scale uncontrolled excessive direct open-air burning will not only cause fires and traffic accidents due to poor visibility, but also damage soil structure and thus cause a huge waste of resources and environmental pollution.

## 5. Conclusions

Taking the beef cattle breeding industry of Jilin Province in China as an example, this paper draws upon the latest emission inventory reports and existing research results, quantifying the GHG emissions of 10 million heads of beef cattle in the ecological cycle model and non-ecological cycle model based on the life cycle assessment. By means of the comparative analysis, we can precisely assess both the negative impact of traditional farming practices and the environmental benefits of the modern beef cattle breeding model.

Firstly, the total GHG emissions from the non-ecological cycle model were approximately four times higher than those from the ecological cycle model, while feed-grain cultivation and manure management were the main emission sources with the most differences in amounts of emissions in both models. In almost all processes of the life cycle assessment, it showed that the GHG emissions of the ecological cycle model were significantly lower than those of its counterpart has. Specially, for the sake of promoting waste and resource recycling in the breeding system, it is worth noting that utilizing straw as livestock feed and manure as fertilizers are core methods of the ecological cycle model.

Secondly, regardless of which kind of GHG emissions are produced in the non-ecological cycle model, the amount of emissions is dramatically higher than that in the ecological cycle model. In particular, the difference in CO_2_ emissions is the largest compared with CH_4_ and N_2_O emissions, and the large proportions of CO_2_ emissions account for 68.01% and 56.17% in their respective breeding systems as well. The ecological cycle model focuses on the combination of farming and breeding, integration of crop and livestock, and green development of agriculture, etc., thus, it gives great prominence to the comprehensive utilization of straw and manure so as to avoid open burning of agriculture waste with much more positive environmental benefits.

Thirdly, in terms of the implementation of the EC model, it makes theoretical sense in Jilin Province that it would have a significant effect on GHG emission reduction in both the planting industry and beef cattle breeding industry; at the same time, it lays the foundation for putting the theory into practice. Most importantly, it will have profound implications for the development of low-carbon agriculture in China, even for other countries with the same characteristics all over the world. Transforming the traditional beef cattle breeding technology into cutting-edge technology of ecological cycle is essential to promoting coordinated development between planting industry and beef cattle breeding industry. In order to develop a sustainable agriculture, we are supposed to strengthen technical supports, reinforce the management and enhance the reuse of straw and manure in the breeding system so as to use fewer resources and therefore generate lower GHG emissions, rather than doing so at the cost of destroying the environment and depleting natural resources through high inputs and outputs. Likewise, the top priority is to adopt the pattern of ecological cycle so that we are able to ensure the improvement of comprehensive production capacity of the beef cattle breeding industry in a resource-saving and environmentally friendly way.

## Figures and Tables

**Table 1:** List of parameters for estimating GHG emissions for the livestock and arable farming system.

Parameters	Data	Unit	Sources
$W_{default}$	319	kg	[52]
$Q_{SN}$	679.33	kg·hm^−2^·a^−1^	[45]
$R_{NF}$	31.05%	-	[45]
$R_{CF}$	53.63%	-	[45]
$w_{c}$	28.41%	-	[53]
$Q_{F}$	22.67	kg·d^−1^	[41]
$ef_{farm-corn}$	1.50	t·t^−1^	[54]
$ef_{ND}$	0.0105	t·t^−1^	[55]
$ef_{NDM}$	0.0105	t·t^−1^	[55]
$ef_{NH}$	0.01	t·t^−1^	[52]
$ef_{NL}$	0.0075	t·t^−1^	[52]
$F_{GAS}$	0.1	t·t^−1^	[52]
$F_{GASM}$	0.2	t·t^−1^	[52]
$F_{L}$	0.25	t·t^−1^	[56]
$F_{LM}$	0.25	t·t^−1^	[56]
$ef_{feed-corn}$	0.0102	t·t^−1^	[57,58]
$R_{burn}$	85.75%	-	[39]
$R_{feed}$	9.88%	-	[39]
G	0.1	-	[5]
$ef_{burn-CO_{2}}$	1.39	kg·kg^−1^	[59,60,61]
$ef_{burn-CH_{4}}$	2.19 × 10^−3^	kg·kg^−1^	[59,60,61]
$ef_{burn-N_{2}O}$	7 × 10^−5^	kg·kg^−1^	[59,60,61]
$VS_{rate,i=1}$	6.8	kg·(1000 kg)^−1^·d^−1^	[52]
$VS_{rate,i=2}$	10.8	kg·(1000 kg)^−1^·d^−1^	[52]
$MS_{i=1}$	28%	-	[52]
$MS_{i=2}$	29%	-	[52]
$ef_{MMC,i=1}$	1.05	kg·kg^−1^	[52]
$ef_{MMC,i=2}$	2.05	kg·kg^−1^	[52]
$ef_{MMDN,i=1}$	0.005	kg·kg^−1^	[52]
$ef_{MMDN,i=2}$	0.01	kg·kg^−1^	[52]
$N_{rate}$	0.41	kg·d^−1^	[52]
$ef_{MMIDN-volatilize}$	0.010	kg·kg^−1^	[52]
$ef_{MMIDN-leach}$	0.011	kg·kg^−1^	[52]
$F_{GAS,i=1}$	0.30	-	[52]
$F_{GAS,i=2}$	0.45	-	[52]
$F_{LEACH,i=1}$	0.035	-	[52]
$F_{LEACH,i=2}$	0.02	-	[52]
$ef_{EF}$	65	kg·head^−1^·a^−1^	[52]

**Table 2:** GHG emissions and proportions of each process.

	Processes	Emission Sources	EC Model (CO_2_-eq, t·a^−1^)	Non-EC Model (CO_2_-eq, t·a^−1^)	Emission Reductions (t·a^−1^)
Planting industry	Corn planting	CO_2_	10.95 × 10^7^ (55.81%)	47.69 × 10^7^ (62.79%)	36.74 × 10^7^
	N-fertilizers application	N_2_O	0.21 × 10^7^ (1.07%)	1.24 × 10^7^ (1.63%)	1.03 × 10^7^
	Manure application	N_2_O	49.53 (-)	22.29 (-)	−27.24
	Feed production	CO_2_	0.07 × 10^7^ (0.36%)	-	−0.07 × 10^7^
	Straw burning	CO_2_	-	3.96 × 10^7^ (5.21%)	3.96 × 10^7^
		CH_4_	-	0.18 × 10^7^ (0.24%)	0.18 × 10^7^
		N_2_O	-	0.05 × 10^7^ (0.07%)	0.05 × 10^7^
Breeding industry	Enteric fermentation	CH_4_	1.82 × 10^7^ (9.28%)	1.82 × 10^7^ (2.39%)	0
	Manure management	CH_4_	6.52 × 10^7^ (33.23%)	20.93 × 10^7^ (27.56%)	14.41 × 10^7^
		N_2_O	0.05 × 10^7^ (0.25%)	0.08 × 10^7^ (0.11%)	0.03 × 10^7^
Total emissions			19.62 × 10^7^ (100%)	75.95 × 10^7^ (100%)	56.33 × 10^7^

Note: (1) The values in parentheses indicate the percentages of total emissions; (2) “(-)” represents extremely small values that should be negligible in this paper; (3) “-” indicates that the process does not exist in the industry; (4) The emission reductions imply GHG emissions of the non-EC model minus the ones of the EC model (same in the below table).

**Table 3:** Proportions of different types of GHG emissions.

Emission Sources	EC Model (CO_2_-eq, t·a^−1^)	Non-EC Model (CO_2_-eq, t·a^−1^)	Emission Reductions (t·a^−1^)
CO_2_	11.02 × 10^7^ (56.17%)	51.65 × 10^7^ (68.01%)	40.63 × 10^7^
CH_4_	8.34 × 10^7^ (42.51%)	22.93 × 10^7^ (30.19%)	14.59 × 10^7^
N_2_O	0.26 × 10^7^ (1.32%)	1.37 × 10^7^ (1.80%)	1.11 × 10^7^
Total emissions	19.62 × 10^7^ (100%)	75.95 × 10^7^ (100%)	56.33 × 10^7^

## Data Availability

Not applicable.

## References

[1] IPCC Climate Change 2022: Mitigation of Climate Change. Working Group III Contribution to the Sixth Assessment Report of the Intergovernmental Panel on Climate Change. https://www.ipcc.ch/site/assets/uploads/2018/02/SYR_AR5_FINAL_full.pdf.

[2] Cavanagh R.D., Trathan P.N., Hill S.L., Melbourne-Thomas J., Meredith M.P., Hollyman P., Krafft B.A., Mc Muelbert M., Murphy E.J., Sommerkorn M. (2021). Utilising IPCC assessments to support the ecosystem approach to fisheries management within a warming Southern Ocean. Mar. Policy.

[3] Minx J.C., Lamb W.F., Andrew R.M., Canadell J.G., Crippa M., Dobbeling N., Forster P.M., Guizzardi D., Olivier J., Peters G.P. (2021). A comprehensive and synthetic dataset for global, regional, and national greenhouse gas emissions by sector 1970–2018 with an extension to 2019. Earth Syst. Sci. Data.

[4] Caro D., Davis S.J., Bastianoni S., Caldeira K. (2014). Global and regional trends in greenhouse gas emissions from livestock. Clim. Change.

[5] FAO Global Livestock Environmental Assessment Model (GLEAM). https://www.fao.org/gleam/results/en/.

[6] Mazzetto A.M., Bishop G., Styles D., Arndt C., Brook R., Chadwick D. (2020). Comparing the environmental efficiency of milk and beef production through life cycle assessment of interconnected cattle systems. J. Clean. Prod..

[7] Lin H.L., Pu Y.F., Ma X.N., Wang Y., Nyandwi C., Nzabonakuze J.D. (2020). The Environmental Impacts of the Grassland Agricultural System and the Cultivated Land Agricultural System: A Comparative Analysis in Eastern Gansu. Sustainability.

[8] New Zealand’s Ministry for the Environment New Zealand’s Greenhouse Gas Inventory 1990–2020. https://environment.govt.nz/assets/publications/GhG-Inventory/New-Zealand-Greenhouse-Gas-Inventory-1990-2020-Chapters-1-15.pdf.

[9] Zhou Z.Z., Tang Y.J., Chi Y., Ni M.J., Buekens A. (2018). Waste-to-energy: A review of life cycle assessment and its extension methods. Waste Manag. Res..

[10] Nwodo M.N., Anumba C.J. (2019). A review of life cycle assessment of buildings using a systematic approach. Build. Environ..

[11] Grant A., Ries R., Kibert C. (2014). Life cycle assessment and service life prediction: A case study of building envelope materials. J. Ind. Ecol..

[12] Takacs B., Borrion A. (2020). The use of life cycle-based approaches in the food service sector to improve sustainability: A systematic review. Sustainability.

[13] Werth S.J., Rocha A.S., Oltjen J.W., Kebreab E., Mitloehner F.M. (2021). A life cycle assessment of the environmental impacts of cattle feedlot finishing rations. Int. J. Life Cycle Assess..

[14] Fiore M., Spada A., Conte F., Pellegrini G. (2018). GHG and cattle farming: CO-assessing the emissions and economic performances in Italy. J. Clean. Prod..

[15] Casey J.W., Holden N.M. (2006). Quantification of GHG emissions from sucker-beef production in Ireland. Agric. Syst..

[16] O’Brien D., Herron J., Andurand J., Care S., Martinez P., Migliorati L., Moro M., Pirlo G., Dolle J.B. (2020). Life beef carbon: A common framework for quantifying grass and corn based beef farms’ carbon footprints. Animal.

[17] Bowman M.S., Soares B.S., Merry F.D., Nepstad D.C., Rodrigues H., Almeida O.T. (2012). Persistence of cattle ranching in the Brazilian Amazon: A spatial analysis of the rationale for beef production. Land Use Policy.

[18] Lynch J., Pierrehumbert R. (2019). Climate Impacts of Cultured Meat and Beef Cattle. Front. Sustain. Food Syst..

[19] Nguyen T.L.T., Hermansen J.E., Mogensen L. (2010). Environmental consequences of different beef production systems in the EU. J. Clean. Prod..

[20] Cederberg C., Stadig M. (2003). System expansion and allocation in life cycle assessment of milk and beef production. Int. J. Life Cycle Assess..

[21] Adrian G.W., Lois P., Sandars D.L. Determining the Environmental Burdens and Resource Use in the Production of Agricultural and Horticultural Commodities. https://www.researchgate.net/publication/265084052_Determining_the_environmental_burdens_and_resource_use_in_the_production_of_agricultural_and_horticultural_commodities_Defra_project_report_IS0205#fullTextFileContent.

[22] Ogino A., Orito H., Shimada K., Hirooka H. (2007). Evaluating environmental impacts of the Japanese beef cow-calf system by the life cycle assessment method. Anim. Sci. J..

[23] Johnson D.E., Phetteplace H.W., Seidl A.F., Schneider U.A., McCarl B.A. Management variations for U.S. beef production systems: Effects on greenhouse gas emissions and profitability. Proceedings of the 3rd International Conference on Methane and Nitrous Oxide Emission Reduction Technology.

[24] Mogensen L., Hermansen J.E., Halberg N., Dalgaard R., Vis J.C., Smith B.G. (2012). Life Cycle Assessment Across the Food Supply Chain. Sustain. Food Ind..

[25] Ridoutt B., Lehnert S.A., Denman S., Charmley E., Kinley R., Dominik S. (2022). Potential GHG emission benefits of *Asparagopsis taxiformis* feed supplement in Australian beef cattle feedlots. J. Clean. Prod..

[26] Ruviaro C.F., Cristiane M.L., Vinicius N.L., Julio O.J.B., Homero D. (2015). Carbon footprint in different beef production systems on a southern Brazilian farm: A case study. J. Clean. Prod..

[27] Newton B.C.J., Tiago C.B., Cassiano E.P., Fabio C.G., Anibal M., Paulo C.F.C. (2019). Public policies for low carbon emission agriculture foster beef cattle production in southern Brazil. Land Use Policy.

[28] Benjamin S., Imke J.M.B., Stwart F.L., Corina E.M. (2021). Reducing greenhouse gas emissions of New Zealand beef through better integration of dairy and beef production. Agric. Syst..

[29] Wang L., Setoguchi A., Oishi K., Sonoda Y., Kumagai H., Irbis C., Inamura T., Hirooka H. (2019). Life cycle assessment of 36 dairy farms with by-product feeding in Southwestern China. Sci. Total Environ..

[30] Vaarst M., Smolders G., Wahome R., Odhong C., Kiggundu M., Kabi F., Nalubwama S., Halberg N. (2019). Options and challenges for organic milk production in East African smallholder farms under certified organic crop production. Livest. Sci..

[31] Cerutti A.K., Contu S., Ardente F., Donno D., Beccaro G.L. (2016). Carbon footprint in green public procurement: Policy evaluation from a case study in the food sector. Food Policy.

[32] Sohoo I., Ritzkowski M., Guo J.Y., Sohoo K., Kuchta K. (2022). Municipal Solid Waste Management through Sustainable Landfilling: In View of the Situation in Karachi, Pakistan. Int. J. Environ. Res. Public Health.

[33] UNCC Greenhouse Gas Inventory Data—Time Series. https://di.unfccc.int/time_series.

[34] Hu Q., Huang H.P., Kung C.C. (2021). Ecological impact assessment of land use in eco-industrial park based on life cycle assessment: A case study of Nanchang High-tech development zone in China. J. Clean. Prod..

[35] Amon B., Cinar G., Anderl M., Dragoni F., Kleinberger-Pierer M., Hortenhuber S. (2021). Inventory reporting of livestock emissions: The impact of the IPCC 1996 and 2006 Guidelines. Environ. Res. Lett..

[36] Green T.R., Kipka H., David O., McMaster G.S. (2018). Where is the USA Corn Belt, and how is it changing?. Sci. Total Environ..

[37] Ort D.R., Long S.P. (2014). Limits on Yields in the Corn Belt. Science.

[38] Tanklevska N., Petrenko V., Karnaushenko A., Melnykova K. (2020). World corn market: Analysis, trends and prospects of its deep processing. Agric. Resour. Econ. Int. Sci. E J..

[39] Na W., Zhao X., Zhu Y., Xi D. (2019). Current situation, problems and countermeasures for comprehensive utilization of main crops straw in Jilin Province. Heilongjiang Agric. Sci..

[40] Sahai S., Sharma C., Singh D.P., Dixit C.K., Singh N., Sharma P., Singh K., Bhatt S., Ghude S., Gupta V. (2007). A study for development of emission factors for trace gases and carbonaceous particulate species from in situ burning of wheat straw in agricultural fields in India. Atmos. Environ..

[41] National Bureau of Statistics of China The First National Pollution Source Census Bulletin. http://www.stats.gov.cn/tjsj/tjgb/qttjgb/qgqttjgb/201002/t20100211_30641.html.

[42] National Bureau of Statistics of China The Second National Pollution Source Census Bulletin. https://www.mee.gov.cn/xxgk2018/xxgk/xxgk01/202006/W020200610353985963290.pdf.

[43] IPCC 2006 IPCC Guidelines for National Greenhouse Gas Inventories. https://www.ipcc-nggip.iges.or.jp/public/2006gl/index.html.

[44] National Bureau of Statistics of China China Statistical Yearbook 2021. http://www.stats.gov.cn/tjsj/ndsj.

[45] Statistic Bureau of Jilin Province Jilin Statistical Yearbook 2021. http://tjj.jl.gov.cn/tjsj/tjnj/2021/ml/indexe.htm.

[46] Fedele A., Mazzi A., Niero M., Zuliani F., Scipioni A. (2014). Can the life cycle assessment methodology be adopted to support a single farm on its environmental impacts forecast evaluation between conventional and organic production? An Italian case study. J. Clean. Prod..

[47] Webb J., Williams A.G., Hope E., Evans D., Moorhouse E. (2013). Do foods imported into the UK have a greater environmental impact than the same foods produced within the UK?. Int. J. Life Cycle Assess..

[48] Weber C.L., Matthews H.S. (2008). Food-miles and the relative climate impacts of food choices in the united states. Environ. Sci. Technol..

[49] Wiedemann S., McGahan E., Murphy C., Yan M.J., Henry B., Thoma G., Ledgard S. (2015). Environmental impacts and resource use of Australian beef and lamb exported to the USA determined using life cycle assessment. J. Clean. Prod..

[50] Crosson P., Shalloo L., O’Brien D., Lanigan G.J., Foley P.A., Boland T.M., Kenny D.A. (2011). A review of whole farm systems models of greenhouse gas emissions from beef and dairy cattle production systems. Anim. Feed Sci. Technol..

[51] IPCC Climate Change 2013—The Physical Science Basis. https://www.ipcc.ch/site/assets/uploads/2018/02/WG1AR5_all_final.pdf.

[52] IPCC 2019 Refinement to the 2006 IPCC Guidelines for National Greenhouse Gas Inventories. https://www.ipcc-nggip.iges.or.jp/public/2019rf/index.html.

[53] Li S., Liu X., He P. (2017). Analyses on nutrient requirements in current agriculture production in China. J. Plant Nutr. Fertil..

[54] Tan Q. (2011). Greenhouse gas emission in China’s agriculture: Situation and challenge. China Popul. Resour. Environ..

[55] Zhang Q., Ju X., Zhang F. (2010). Re-estimation of direct nitrous oxide emission from agricultural soils of China via revised IPCC2006 guideline method. Chin. J. Eco-Agric..

[56] Zhao R., Chen X., Zhang F. (2009). Nitrogen cycling and balance in winter-wheat-summer-maize rotation system in northern China plain. Acta Pedol. Sin..

[57] Parks N. (2007). Livestock’s long shadow. Front. Ecol. Environ..

[58] Goodland R. (2013). Correspondence: Lifting livestock’s long shadow. Nat. Clim. Change.

[59] Li X.G., Wang S.X., Duan L., Hao J., Li C., Chen Y.S., Yang L. (2007). Particulate and trace gas emissions from open burning of wheat straw and corn stover in China. Environ. Sci. Technol..

[60] Zhang H.F., Ye X.N., Cheng T.T., Chen J.M., Yang X., Wang L., Zhang R.Y. (2008). A laboratory study of agricultural crop residue combustion in China: Emission factors and emission inventory. Atmos. Environ..

[61] Andreae M.O. (2019). Emission of trace gases and aerosols from biomass burning—An updated assessment. Atmos. Chem. Phys..

[62] Hatew B., Bannink A., Laar H., Jonge L.H., Dijkstra J. (2016). Increasing harvest maturity of whole-plant corn silage reduces methane emission of lactating dairy cows. J. Dairy Sci..

[63] Hayashi K. (2013). Practical recommendations for supporting agricultural decisions through life cycle assessment based on two alternative views of crop production: The example of organic conversion. Int. J. Life Cycle Assess..

[64] Iqbal J., Mitchell D.C., Barker D.W., Miguez F., Sawyer J.E., Pantoja J., Castellano M.J. (2015). Does nitrogen fertilizer application rate to corn affect nitrous oxide emissions from the rotated soybean crop?. J. Environ. Qual..

[65] Chadwick D., Sommer S.G., Thorman R., Fangueiro D., Cardenas L., Amon B., Misselbrook T. (2011). Manure management: Implications for greenhouse gas emissions. Anim. Feed. Sci. Technol..

[66] Cao G.L., Zhang X.Y., Wang Y.Q., Zheng F.C. (2008). Estimation of emissions from field burning of crop straw in China. Chin. Sci. Bull..

[67] Zhang X.H., Lu Y., Wang Q.G., Qian X. (2019). A high-resolution inventory of air pollutant emissions from crop residue burning in China. Atmos. Environ..

